# Cellular Model of Warburg Effect Identifies Tumor Promoting Function of UCP2 in Breast Cancer and Its Suppression by Genipin

**DOI:** 10.1371/journal.pone.0024792

**Published:** 2011-09-15

**Authors:** Vanniarajan Ayyasamy, Kjerstin M. Owens, Mohamed Mokhtar Desouki, Ping Liang, Andrei Bakin, Kumarasamy Thangaraj, Donald J. Buchsbaum, Albert F. LoBuglio, Keshav K. Singh

**Affiliations:** 1 Department of Cancer Genetics, Roswell Park Cancer Institute, Buffalo, New York, United States of America; 2 Department of Pathology and Laboratory Medicine, Medical University of South Carolina, Charleston, South Carolina, United States of America; 3 Department of Biological Sciences, Brock University, St. Catharines, Ontario, Canada; 4 Centre for Cellular and Molecular Biology, Hyderabad, India; 5 Department of Radiation Oncology, University of Alabama at Birmingham, Birmingham, Alabama, United States of America; 6 University of Alabama at Birmingham Comprehensive Cancer Center, University of Alabama at Birmingham, Birmingham, Alabama, United States of America; 7 Departments of Genetics, Pathology, Environmental Health, Center for Free Radical Biology, Center for Aging and University of Alabama at Birmingham Comprehensive Cancer Center, University of Alabama at Birmingham, Birmingham, Alabama, United States of America; University of Florida, United States of America

## Abstract

The Warburg Effect is characterized by an irreversible injury to mitochondrial oxidative phosphorylation (OXPHOS) and an increased rate of aerobic glycolysis. In this study, we utilized a breast epithelial cell line lacking mitochondrial DNA (rho^0^) that exhibits the Warburg Effect associated with breast cancer. We developed a MitoExpress array for rapid analysis of all known nuclear genes encoding the mitochondrial proteome. The gene-expression pattern was compared among a normal breast epithelial cell line, its rho^0^ derivative, breast cancer cell lines and primary breast tumors. Among several genes, our study revealed that over-expression of mitochondrial uncoupling protein UCP2 in rho^0^ breast epithelial cells reflects gene expression changes in breast cancer cell lines and in primary breast tumors. Furthermore, over-expression of UCP2 was also found in leukemia, ovarian, bladder, esophagus, testicular, colorectal, kidney, pancreatic, lung and prostate tumors. Ectopic expression of UCP2 in MCF7 breast cancer cells led to a decreased mitochondrial membrane potential and increased tumorigenic properties as measured by cell migration, in vitro invasion and anchorage independent growth. Consistent with in vitro studies, we demonstrate that UCP2 over-expression leads to development of tumors in vivo in an orthotopic model of breast cancer. Genipin, a plant derived small molecule, suppressed the UCP2 led tumorigenic properties, which were mediated by decreased reactive oxygen species and down-regulation of UCP2. However, UCP1, 3, 4 and 5 gene expression was unaffected. UCP2 transcription was controlled by SMAD4. Together, these studies suggest a tumor-promoting function of UCP2 in breast cancer. In summary, our studies demonstrate that i) the Warburg Effect is mediated by UCP2; ii) UCP2 is over-expressed in breast and many other cancers; iii) UCP2 promotes tumorigenic properties in vitro and in vivo and iv) genipin suppresses the tumor promoting function of UCP2.

## Introduction

Mitochondria play a central role in the cell growth, metabolism and cell death. Mitochondria produce energy by oxidative phosphorylation (OXPHOS) and are involved in the metabolism of fatty acids, nucleotides, amino acids and carbohydrates; synthesis of heme, Fe-S, ubiquinone and cofactors; DNA replication, repair and methylation; and antibacterial defense [1], [2], [3], [4]. Since mitochondria perform multiple cellular functions, defective mitochondria contribute to a vast number of human diseases [5], [6], [7], [8].

Otto Warburg in 1956 proposed that cancer was caused by defects in mitochondria, forcing cells to shift to energy production through glycolysis despite aerobic conditions [9]. This characteristic of cancers is described as the “Warburg Effect.” The Warburg Effect plays an important role in tumor development by remodeling the metabolic profile, which allows tumor cell survival under adverse conditions [10], [11], [12], [13], [14]. Warburg stated that cancer cells originate in two phases: i) “The first phase is the irreversible injury to respiration (OXPHOS).” ii) “The irreversible injury to respiration (OXPHOS) is followed, by a long struggle for existence by the injured cells to maintain their structure, in which a part of the cells perish (apoptosis) for lack of energy, while another part succeed in replacing the lost respiration energy by aerobic glycolysis” [9]. Our studies and those conducted by others suggest that the underlying cause of “irreversible injury” to OXPHOS includes reduced mtDNA content and mutations in mtDNA and in nuclear genes affecting OXPHOS [15], [16], [17], [18]. Our previous studies have also revealed that defects in OXPHOS induce a “*mitocheckpoint*” response involving epigenetic and genetic changes in the nuclear genome [3], [19], [20], [21]. We reported an undetectable level of mtDNA-encoded cytochrome c-oxidase subunit II in more than 40% of breast and ovarian tumors, suggesting a significant depletion of mtDNA in primary tumors [22], [23]. Other laboratories have also described a decrease in mtDNA content in breast [24], [25], renal [26], hepatocellular [27], gastric [28] and prostate tumors [16]. Depletion of mtDNA is also proportional to a decrease in OXPHOS levels in renal tumors [29]. A reduced mtDNA copy number is also associated with resistance to apoptosis and increased metastasis [30], [31], [32].

We recently developed a breast epithelial cell line devoid of mitochondrial DNA (rho^0^) that recapitulates the Warburg Effect [33] and mimics depletion of mtDNA in the variety of cancers described above. The rho^0^ cells lack mtDNA and thus lack the critical subunits of the respiratory chain, causing irreversible injury to respiration and forcing the cells to utilize aerobic glycolysis for ATP production [33], [34], [35]. The rho^0^ cells exhibiting the Warburg Effect serve as a valuable tool for identifying genomic and epigenomic changes associated with tumorigenesis [33], [36]. In this paper, we determined whether the gene expression changes associated with rho^0^ state in epithelial cells reflect changes in cancer cell lines and in primary tumors. Among many genes, we confirmed that UCP2 was over-expressed in rho^0^ epithelial cells, breast cancer cell lines and primary breast tumors. UCP2 is a member of the family of uncoupling proteins located in the inner mitochondrial membrane [37]. UCP2 function is linked to obesity and diabetes [38]. The role of UCP2 in cancers is not well understood. This paper describes the tumor promoting properties of UCP2 in vitro and in vivo in a mouse xenograft model. We also describe that genipin, a small molecule extracted from the gardenia plant, reduces the tumor promoting properties induced by over-expression of UCP2.

## Materials and Methods

### MitoExpress array design

All proteins related to mitochondrial structure, function and dynamics were extracted from the published literature [2], [39], [40], [41], [42] and from various public databases, such as the Human Mitochondrial Protein Database, Mitomap, Mitop2, Migenes and Mitoproteome. Human orthologs from the mitochondrial proteins of lower organisms were also extracted from the NCBI, and redundant genes were carefully removed. The corresponding probe sets of genes were selected from NetAffx. The extracted mitochondrial proteins were made into a single list and then filtered to get rid of redundancy. Probe sets corresponding to mitochondrial proteins selected for analysis were identified from the list of human expression chip HG-U133. Probe sets of the mitochondrial genes, along with the standard human normalization probes, were tiled on a chip using a unique combination of photolithography and combinatorial chemistry in the 11 micron, 100–2187 affymetrix array format. The final set of genes (1,136 genes) tiled on the mitochondrial expression chip is given in Table S1.

### Cell culture

All the cell lines were purchased from ATCC (Manassas, VA). The breast epithelial cells (MCF12A) were grown in media consisting of Ham's F12/Dulbecco's Modified Eagle's Media (DMEM-F12; Cellgro, Herndon, VA) containing 100 U/ml penicillin and 100 µg/ml streptomycin (Invitrogen, Carlsbad, CA), 100 ng/ml cholera toxin (Invitrogen), 0.5 µg/ml hydrocortisone (Sigma, St Louis, MO), 10 µg/ml insulin (Sigma), 20 ng/ml EGF (Sigma) and 10% horse serum (Invitrogen); the mtDNA-compromised breast epithelial cell line (MCF12A rho^0^) was cultured in MCF12A medium with 50 µg/ml uridine (Sigma), and breast cancer cell lines (MCF7, MDAMB231, MDAMB435) were grown in Dulbecco’s Modified Eagle’s Media (DMEM; Cellgro) supplemented with 10% fetal bovine serum (FBS; Cellgro), 100 U/ml penicillin and 100 µg/ml streptomycin. Cells were maintained in a 37°C, 5% CO_2_ environment. Mouse mammary epithelial NMuMG cells were cultured at 37°C under an atmosphere of 5% CO_2_ in DMEM supplemented with 10% FBS (Cellgro) and 10 µg/ml insulin.

### Transfection and selection of stable cell lines

The UCP2 cDNA was kindly provided by Dr. Mills. The plasmid was transfected into MCF7 cells with Fugene HD transfection reagent (Roche, Fishers, IN) and stably transfected cells were selected for after 48 hrs with medium containing G418 (2 mg/ml). UCP2 over-expression was confirmed by Western blot analysis. For the Smad4 silencing, NMuMG cells were transfected with siRNA duplexes to Smad4 (Dharmacon Inc., Lafayette, CO) and scramble control siRNA labeled with rhodamine (Qiagen, Valencia, CA) using Oligofectamine reagents (Invitrogen). The silencing of Smad4 was confirmed by mouse monoclonal antibodies to Smad2/3 and Smad4 (BD Biosciences, Palo Alto, CA).

### RNA isolation and cDNA synthesis

Total RNA was isolated from human breast cell lines and mouse NMuMG cells using the Trizol method (Invitrogen) and further purified by RNeasy Midi Columns (Qiagen). The RNA was quantified by Nanodrop (Nanodrop Technologies, Wilmington, DE), and quality was checked by O.D. at 260 nm. The primary breast tumor RNA was obtained from the Pathology Core Facility at Roswell Park Cancer Institute. cDNA was synthesized with a SuperScript III First Strand Synthesis kit, using the standard protocol (Invitrogen). For microarray experiments, the cDNA was synthesized according the manufacturer’s guidelines.

### Gene-expression analysis

7 µg of total RNA was used for the synthesis of double-stranded cDNA with the Genechip tiling WT double-stranded cDNA synthesis kit (Affymetrix, Santa Clara, CA). After the cDNA synthesis, the samples were cleaned up by the sample clean-up module and used for chip analysis and real-time PCR confirmation. The doubled-stranded DNA was fragmented and labeled with a Genechip WT double-stranded DNA terminal labeling kit. The labeled DNA was hybridized along with the control oligonucleotide B2 to the arrays. The arrays were then washed and stained according to the manufacturer’s protocol. The arrays were scanned by the Affymetrix scanner and viewed by the microarray suite. The data was exported to Array Assist (Stratagene) and analyzed. We used the PLIER algorithm to normalize the data and generate raw signal values. Background noise was filtered out by using the detection above background. Once the raw signal values were generated, they were filtered for genes that were ≥two-fold and had a p-value ≤0.05. A principal component analysis was done to ensure that samples clustered correctly. All data is MIAME compliant and the raw data has been deposited in a MIAME-compliant database GEO, as detailed by the MGED Society.

A mouse microarray analysis was performed using Affymetrix MOE430_2AB chips. Total RNA samples were isolated from NMuMG cells transfected with siRNA control or siSmad4 duplexes, following treatment with 2 ng/ml TGF-β1 (R&D Systems, Minneapolis, MN) for 24 h. The Affymetrix gene-expression data in CEL format was processed and converted to numeric data by the gcRMA method using the ExpressionFileCreator module of the GenePattern package [43], during which quantile normalization and computation of “present” and “absent” calls were included. To identify differentially expressed genes, we used criteria requiring a minimal of a twofold change between the compared experiments and a probe showing higher expression to have a minimal value of 75 and shown as “present.” The latter two parameters reduced false positives resulting from low expression and/or inconsistent probe measurements. The probes showing up- and down-regulation in response to TGF-β treatment were collected from NMuMG and siSmad4-transfected cells. Overlaps between the lists were identified by in-house PERL scripts. The function classification and statistical over-representation of gene-function categories (e.g., gene ontology terms) were analyzed using the DAVID package [44].

### Confirmation of differentially expressed genes

Among the differentially expressed genes, highly up- and down-regulated genes were selected for validation based on their relevance to mitochondrial structure and function. Confirmation of the differential expression was done by Superarray RT-PCR plates (SA Biosciences, Frederick, MD). The optimized primers for the selected genes were coated to the custom-made 96-well plate PCR array. Quantitative real-time PCR assays were performed using ABI Prism 7900 (Applied Biosystems, Foster City, CA) with SYBR GreenER PCR Master Mix (Invitrogen), according to the manufacturer’s protocol. The data was analyzed by RQ Manager (Applied Biosystems) software, and the relative gene expression of the genes was analyzed by comparing results to the housekeeping gene (β-actin) using the Delta delta Ct method.

### Western blot analysis of proteins

Western blot was used to check the over-expression of UCP2 protein. The whole cell extract was prepared using the standard methods, and 100 µg of protein was loaded and resolved on 12% SDS-PAGE. The separated proteins were transferred to an Immuno-Blot PVDF Membrane (Millipore, Billerica, MA), followed by incubation for one hour at room temperature with 5% milk to block nonspecific binding. Blots were incubated overnight at 4°C with 1∶300 UCP2 (Santa Cruz Biotechnology, Santa Cruz, CA) antibody, followed by washing and incubation with a 1∶5000 anti-goat IgG antibody (Vector Laboratories, Burlingame, CA). The bound secondary antibody was detected using an enhanced chemiluminescence solution. The membranes were exposed to CL-XPosure film (ThermoScientific, Rockford, IL). Anti-mouse Complex III core 2 subunit antibody (Molecular Probes, Eugene, OR) was used to determine equal protein-loading.

### Immunohistochemistry

All experiments were approved by the Roswell Park Cancer Institute Institutional Review Board, permit number I92106. A tissue-array slide from the Cooperative Human Tissue Network (CHTN) and Tissue Array Research Program (TARP5) of the National Cancer Institute, National Institutes of Health, Bethesda, MD, was also used in the present study. Consent from patients was not needed, as anonymous tissue samples were used for study. These samples were collected by the biorepository resource facility of the Roswell Park Cancer Institute and provided to us under IRB-approved permit number I92106. The slide contained breast and ovarian carcinomas as well as multiple benign tissues from different organs. One section from a formalin-fixed, paraffin-embedded benign spleen was used as a positive control. Characterization of the lesions and grading of the tumors was done by a pathologist, as previously described [23]. The immunohistochemistry protocol, as described by Desouki *et al.*, 2005 [23], was applied with modifications. Briefly, the slides were de-paraffinized by incubation in xylene and ascending grades of alcohol. Antigen retrieval was done by heating in a citrate-based, antigen-unmasking solution (Vector Laboratories, Burlingame, CA, cat. no. H-3300) for 30 min at 98°C, incubated in 3% hydrogen peroxide (H_2_O_2_) for 10 min, blocked with blocking peptide for 30 min, incubated with 8 µg/ml anti-UCP1 (Abnova, Walnut, CA) and anti-UCP2 (Santa Cruz Biotech, Santa Cruz, CA, cat # sc-6525) antibody for 1 h at room temperature, followed by incubation with biotinylated secondary anti-rabbit and anti-goat solution for 30 min, respectively, and another 30 min with Vectastatin ABC kit (Vector Laboratories, Burlingame, CA). The color was developed by incubating slides with peroxidase substrate solution, followed by counterstaining with Hematoxylin. The sections were also incubated with secondary antibody only to check nonspecific bindings. All sections were examined with an Olympus BX50 microscope, and pictures were taken with an Olympus DP 70 connected to DP Controller software (Olympus, Center Valley, PA).

An Epi-Info software program, version 3.5.1, was used for statistical analysis. A linear correlation test was performed to determine the correlation between tumor grades and UCP2 immunoreactivity. Scoring of immunoreactivity was considered to be negative or positive, with the same parameters described above (score + <10% positive, score ++ 10–50% positive and score +++ >50% positive) [23]. Grades and IHC scores were considered nominal to calculate the correlation coefficient.

### Mitochondrial membrane potential and ATP measurements

Mitochondrial membrane potential was measured by the fluorescence of tetramethylrhodamine, ethyl ester (TMRE, Molecular Probes, Eugene, OR). Cells were incubated with 100 nM TMRE for 35 min, harvested and resusupended in PBS. The fluorescence of the cells was read on the FL2 channel of a Becton Dickinson FACScan Flow Cytometer (Franklin Lakes, NJ) [45]. Intracellular ATP levels were measured using the ATP bioluminescent assay kit (Sigma) according to the manufacturer’s protocol.

### MTT assay

Cell viability and proliferation were measured by rinsing the cells with PBS, then incubating them with 833 µg/ml of 3-(4,5-dimethlythiazol-2-yl)-2,-diaphenyltetrazolium bromide (MTT) (Molecular Probes) for 3 h. MTT formazan crystals were dissolved in an isopropanol solution containing 4 mM HCl and 0.1% Nonidet P-40. The absorbance of the reduced purple formazan was measured at 590 nm with a reference wavelength of 620 nm [46].

### Wound-healing

Cell migration was measured by a wound-healing scratch assay. Scratches were made in a confluent monolayer of cells with a sterile pipette tip and immediately photographed. Migration of cells into the scratch was monitored for up to 24 h [47].

### Matrigel invasion

In vitro invasion of cells was measured by Matrigel invasion using a Matrigel Boyden chamber (BD Biosciences, Bedford, MA). Cells were serum-starved for 4 h, and seeded in the upper chamber in DMEM with 0.1% BSA. NIH-3T3-conditioned media containing 10% FBS was added to the bottom as a chemo-attractant. Cells were allowed to migrate for 24 h, and then the membrane was stained with a Diff-Quik Stain Set (Dade Behring, Newark, DE) [18]. Cells were also plated in control inserts without any matrigel (BD Biosciences) to control for toxicity and adhesion to the membrane.

### Soft-agar assay

Anchorage-independent growth was measured by a colony formation in soft-agar. Cells were mixed with 0.3% agarose and plated on dishes containing 0.5% agarose. Growth media was added to each well and replenished every 3 to 4 days. Colonies were visualized by staining with 0.005% crystal violet for 1 h [48].

### Orthotopic tumor growth of MCF-7 and MCF-7-UCP2 cells

Female athymic nude mice were obtained at 4-6 weeks of age from Harlan Laboratories (Indianapolis, IN). 17β-estradiol pellets (NE-121, 1.7 mg, 90 day release, Innovative Research of America, Sarasota, FL), were implanted subcutaneously two days prior to tumor cell injection. Orthotopic breast cancer xenografts were established by implanting 1×10^7^ MCF-7 or MCF-7-UCP2 cells in a 1∶1 mixture with Matrigel (BD Biosciences, San Jose, CA) into the mammary fat pad in groups of 10 mice each. Tumor growth was monitored twice weekly by measuring tumor diameter in the two largest dimensions with calipers. Mean tumor size was calculated from the product of individual tumor diameters and reported relative to tumor size at day 3 post-tumor cell injection. All studies were conducted in accordance with University of Alabama at Birmingham Institutional Animal Care and Use Committee regulations.

### Clonogenic survival

Reproductive viability was assayed by clonogenic survival. Cells were harvested after treatment and plated at a low density. Colonies were allowed to grow for two weeks and were then fixed with 70% ethanol and stained with 0.1% Coomassie brilliant blue stain (BioRad).

### Reactive oxygen species measurement

Cells were analyzed for reactive oxygen species (ROS) production by labeling with 10 µM dihydroethidium (DHE) (Molecular Probes) for 40 min. Oxidation of DHE was analyzed on a FACSCalibur flow cytometer (Becton Dickinson). 10,000 events were collected for each sample. ROS levels were expressed as mean fluorescence intensity (MFI), which was calculated by WinList software (Verity Software House).

### Genipin treatment

Genipin is a chemical compound that has been documented to inhibit UCP2 expression and activity. MCF12A, MCF7 and UCP2 over-expressing cells were plated in 96-well plates and treated with varying doses of genipin (0–50 µM) for 48 h, and the cell viability was analyzed by MTT assay, as described above. For all other assays, cells were treated with 0, 5 and 10 uM genipin. A clonogenic survival assay was done to study the effect of genipin on the survival of the UCP2 over-expressing cells. A RT PCR was done to check whether any other member of the UCP family was affected by the genipin treatment. The functional significance of genipin on the UCP2 over-expressing cells was studied by a wound-healing, matrigel invasion. DHE oxidation of the MCF7 cells was also analyzed to see the impact of genipin treatment.

### *In silico* analysis

UCP2 expression was checked with the Oncomine database [49]. The higher expression of UCP2 was analyzed between the normal and corresponding tumors. Similarly, UCP2 was analyzed among multiple cancers, such as bladder, colorectum, kidney, liver, lung, ovarian, pancreatic and prostate cancers.

## Results

### MitoExpress analyses

We developed an oligonucleotide array named MitoExpress, contracted through Affymetrix, Inc., to study the differential expression of the nuclear genes encoding the mitochondrial proteome in breast epithelial cells, its rho^0^ derivative and primary breast cancer cells. MitoExpress contains 1,136 nuclear genes encoding mitochondrial proteins that were extracted from various databases and published literature and then filtered to remove redundant genes (Table S1). It also contains standard probe sets for 146 housekeeping genes, the same as that of human expression chip HG-U133 for normalization and background correction.

### Gene-expression similarity in rho^0^ breast epithelial and breast cancer cell lines

**Figure 1:**
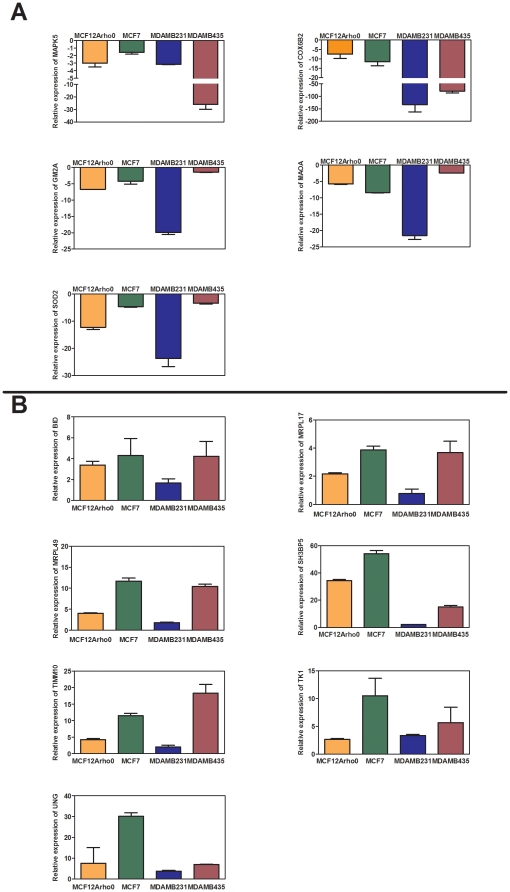
Changes in gene expression due to OXPHOS defect in breast epithelial cell line. (A) Down-and (B) up-regulated genes in rho^0^ and breast cancer cell lines. MitoExpress chip data was validated by quantitative real time PCR (RT-PCR). Fold changes were calculated relative to the parental MCF12A rho^+^ using beta actin as control.

Our previous study suggests that depletion of mitochondrial DNA in breast epithelial cells leads to tumorigenic transformation in vivo [33]. We hypothesized that the pattern of gene expression changes in rho^0^ breast epithelial cells may help identify novel tumor-suppressor and oncogenes associated with irreversible injury to OXPHOS, as described by Warburg [50]. Using the MitoExpress array, we therefore analyzed gene-expression changes in the MCF12A parental breast epithelial cell line and its rho^0^ derivative. The changes in gene expression that were observed in the rho^0^ cells were compared to expression in the breast cancer cell lines. The gene expression that was twofold or lower compared to the parental MCF12A rho^+^ cell line was considered to be down-regulated. We found that a total of 29 genes were down-regulated in both the rho^0^ and breast cancer cell lines (Table S2). The gene expression that was twofold or higher compared to the parental MCF12A cell line was considered to be up-regulated. A total of 37 genes were up-regulated in both the rho^0^ epithelial cells and breast cancer cell lines (Table S3). The differentially expressed genes are described to be involved in critical functions such as cellular metabolism, apoptosis, transport, translation, DNA replication and repair, splicing factor and cell redox homeostasis. The key genes from each set of commonly down- and up-regulated groups from the rho^0^ epithelial cell line and breast cancer cell lines were then validated (see Tables S2 and S3). These genes were analyzed on a 96-well plate pre-coated with primers for quantitative real-time PCR (RT-PCR) (custom made by Super Arrays) using the SYBR green method. We confirmed down regulation of five selected genes (COX6B2, MAPK5, GM2A, SOD2, and MAOA) in rho^0^ cells (Figure 1A). Among these validated genes, we compared expression in breast cancer cell lines to normal breast epithelial cells. As hypothesized, we found that selected genes were also down-regulated in breast cancer cell lines (Figure 1A). Likewise, we confirmed the up-regulation of seven genes (BID, MRRL17, SH3PB5, MRRL49, TK1, TIMM10, and UNG) in rho^0^ cells and breast cancer cell lines (Figure 1B). We conclude that an OXPHOS defect in rho^0^ breast epithelial cells mimics the gene expression changes of nuclear DNA-encoded mitochondrial proteins as seen in breast cancer cell lines.

### Cytochrome C oxidase gene family is down-regulated in breast cancer

**Figure 2:**
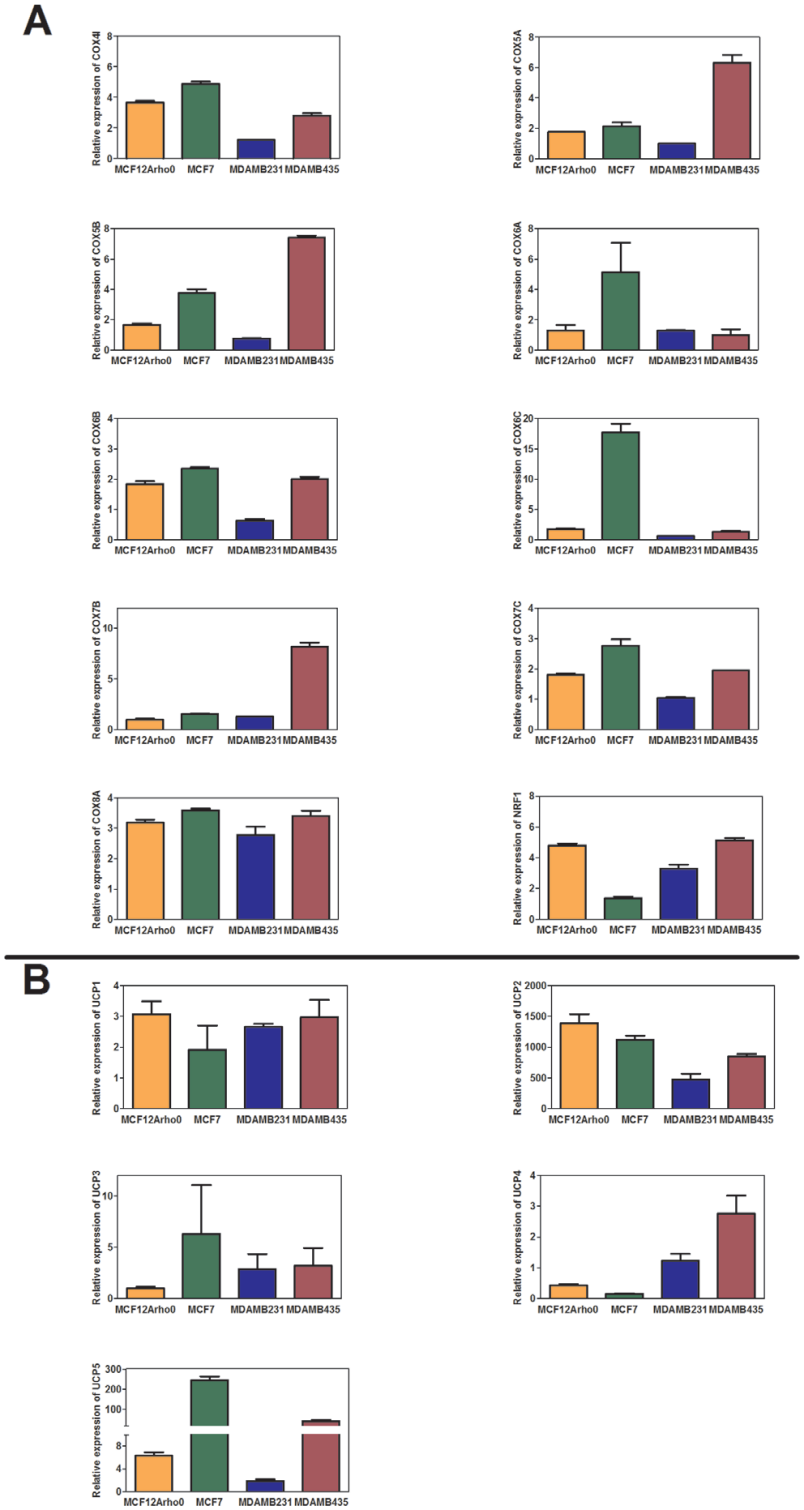
Gene expression profile of COX and UCP family members. Expression profile of (A) COX family members, and (B) UCP family members in rho^0^ and breast cancer cell lines relative to the parental MCF12A rho^+^.

Our initial gene expression comparisons identified COX6B2 as being down regulated in rho^0^ and breast cancer cells. This is of particular interest because COX6B2 is a subunit of cytochrome oxidase (COX). COX is the terminal enzyme of OXPHOS that reduces molecular oxygen to water. It is composed of 10 nuclear-encoded and three mitochondrially encoded subunits [51]. We measured changes in expression in all of the nuclear-encoded members of the COX family, as well as the transcription factor and COX master regulator, NRF1. The pattern of expression in rho^0^ cells and among breast cancer cell lines was similar in that the genes encoding the COX protein complex, as well as the COX master regulator NRF1, were down-regulated (Figure 2A).

### UCP gene family members are up-regulated in breast cancer

In addition to the seven up-regulated genes described above, we found that UCP2 (**u**n**c**oupling **p**rotein 2) was expressed several-hundred-fold in rho^0^ breast epithelial cells and breast cancer cell lines. UCP2 belongs to a family of proteins that uncouples mitochondrial respiration and ATP synthesis. To date, five members of the UCP family have been described [53]-[60]. We measured the expression of all five members of the UCP family *viz.* UCP1 to 5. We confirmed the large increase in UCP2 expression and also found a significant increase in gene expression of UCP1 and 5 in the rho^0^ cells and breast cancer cell lines (Figure 2B).

### Increased expression of UCP1 in primary breast tumors

**Figure 3:**
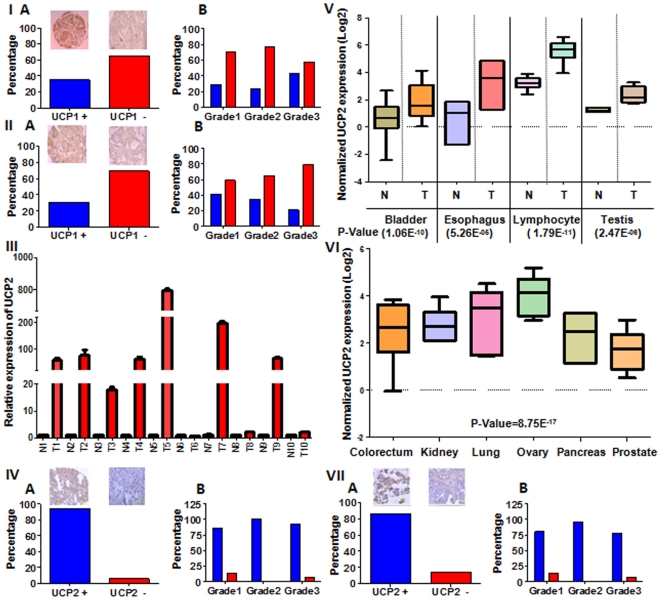
Expression of UCP family members in primary tumors. (I-A) UCP1 expression in breast tumors. (I-B) UCP1 expression in relation to breast tumor grade. (II-A) UCP1 over-expression in ovarian tumors. (II-B) UCP1 expression in relation to ovarian tumor grade. (III) UCP2 expression at RNA level in matched normal and primary breast tumors. (IV-A) UCP2 expression at protein level in breast-tumors. (IV-B) UCP2 expression in relation to breast tumor grade. (V) Oncomine data showing UCP2 expression in normal (N) Vs tumor (T) of bladder, esophagus, lymphocyte, testis. The p-value is given in brackets underneath each tumor type. (VI) Oncomine data showing UCP2 over-expression in colorectal, kidney, lung, pancreatic, prostate and ovarian tumors. (VII-A) Validation of UCP2 over-expression in primary ovarian tumors. (VII-B) Lack of correlation between UCP2 expression and ovarian tumor grade.

UCP1 was the first identified member of the UCP family [52]-[59]. We found a significant increase in UCP1 expression in breast cancer cell lines, so we analyzed UCP1 expression in human breast carcinomas. A TARP5 slide with several breast carcinoma sections from different cases helped us to screen a relatively large number of cases. Immunohistological examination showed a high expression of UCP1in 35% of breast tumors (Figure 3-IA). There was no correlation of UCP1 expression with the tumor grade (Figure 3-IB). We expanded UCP1 expression in ovarian carcinoma and found 31% of them showed high expression (Figure 3-IIA). As with breast tumors, there was no correlation between UCP1 expression and tumor grade (Figure 3-IIB). We conclude that UCP1 is up-regulated in breast cancer cell lines and primary breast as well as other tumors.

### UCP2 is over-expressed in primary breast tumors

The above studies identified that UCP2 expression was increased several-hundred-fold in rho^0^ breast epithelial cells and cancer cell lines. We therefore focused our study on UCP2. We measured UCP2 expression by real-time PCR in matched normal and primary breast tumors and found that seven out of 10 tumors showed a significant increase in UCP2 expression (Figure 3-III). We used a TARP5 slide to screen a breast carcinoma for UCP2 expression. Examination of the breast-tumor sections revealed that 94% of breast carcinoma cases showed a high level of UCP2 expression (Figure 3-IVA). Our analysis revealed no correlation between tumor grade and UCP2 expression (r = 0.01) (Figure 3-IVB). Eighty-six percent of grade 1, 100% of grade 2 and 93% of grade 3 breast carcinomas were positive for UCP2.

### UCP2 is over-expressed in multiple cancers

Since the Warburg Effect relates not only to breast cancer but is a general phenomenon found in cancers [7], we investigated the expression of UCP2 in other cancers. Our analyses of Oncomine data sets revealed that UCP2 is over-expressed in ovarian, bladder, esophageal, testicular, kidney, colorectal, lung, pancreas and prostate cancers as well as in leukemia (Figure 3-V). As a test of Oncomine data, we chose to validate UCP2 over-expression in ovarian cancers (Figure 3-VI). Examination of UCP2 expression in primary ovarian tumors indeed revealed that approximately 90% of ovarian carcinoma cases expressed UCP2 at a very high level (Figure 3-VIIA). No correlation was found between tumor grade and UCP2 expression (r = 0.0) (Figure 3-VIIB). Eighty percent of grade 1, 96% of grade 2 and 78% of grade 3 ovarian tumors strongly expressed UCP2 in more than 10% of cells (IHC score 2 and 3). We conclude that UCP2 over-expression is a general phenomenon linked to the Warburg Effect in cancer.

### Ectopic expression of UCP2 enhances tumorigenic properties in vitro and promotes tumor growth in vivo

**Figure 4:**
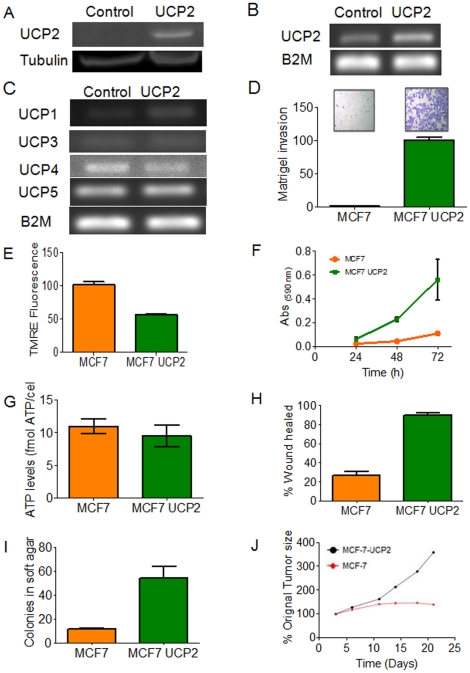
UCP2-induced tumor promoting properties in vitro and tumor growth in vivo. (A) Western blot analysis showing the over-expression of UCP2 at protein level. (B) RT-PCR showing over-expression of the UCP2 at transcript level. (C) RT-PCR showing expression of UCP family members in the UCP2 over-expressing cells. (D) TMRE fluorescence of the UCP2 over-expressing cells (E) ATP production in the UCP2 over-expressing cells. (F) Proliferation of UCP2 over-expressing cells. (G) Wound healing in the UCP2 over-expressing cells. (H) Matrigel invasion of the UCP2 over-expressing cells. (I) Soft agar assay of the UCP2 over-expressing cells. (J) Growth of MCF-7 parental and MCF-7-UCP2 orthotopic xenografts in athymic nude mice. MCF-7 parental or MCF-7-UCP2 over-expressing cells were injected into the mammary fat pad two days after subcutaneous implantation of 17β-estradiol pellets. Tumor size averaged 36 mm^2^ at 3 days post-tumor cell injection in each group. N = 10 mice/group.

The above studies demonstrated an over-expression of UCP2 in a variety of primary tumors. Therefore, in order to define the role of UCP2 in cancer, we over-expressed UCP2 in MCF7 breast cancer cells. The over-expression of UCP2 was confirmed by Western blot and RT-PCR analyses (Figures 4 A and B). UCP2 ectopic expression did not result in significant changes in expression of other UCP members (Figure 4C). The membrane potential of UCP2 over-expressing cells was decreased by about 40% (Figure 4D). However, ATP production remained unchanged (Figure 4E).

To test the effect of UCP2 over-expression on the tumorigenic phenotype, we conducted in vitro assays, including cell proliferation, wound-healing, matrigel invasion and growth in soft agar. Figure 4F demonstrates that UCP2 over-expressing cells proliferate faster than control cells. The doubling time of UCP2 over-expressing cells was 15.2 h compared to 22.3 h for parental MCF7 cells expressing vector only. Wound-healing measurements also showed faster cell migration in the UCP2 over-expressing cells (Figure 4G). We measured in vitro invasion by matrigel assay. The UCP2 over-expressing cells showed a high rate of invasion in the matrigel as compared to the control cells (Figure 4H). Furthermore, the UCP2 over-expressing cells showed an increase in anchorage independent growth as assayed by colony formation in the soft agar assay (Figure 4I).

In addition to the in vitro tumorigenic phenotype, we measured tumor growth in the mouse xenograft model. The growth of MCF-7 and MCF-7-UCP2 orthotopic tumors in athymic nude mice is shown in Fig. 4J. The MCF-7-UCP2 tumors grew much more rapidly than MCF-7 tumors, with a 300% increase in MCF-7-UCP2 tumor size at day 19 as compared to the average size at 3 days post-transplant (36 m^2^), in comparison to a 125% increase for MCF-7 tumors. Together, these studies demonstrate that over-expression of UCP2 promotes tumorigenicity.

### Genipin suppresses tumor promoting properties of UCP2

**Figure 5:**
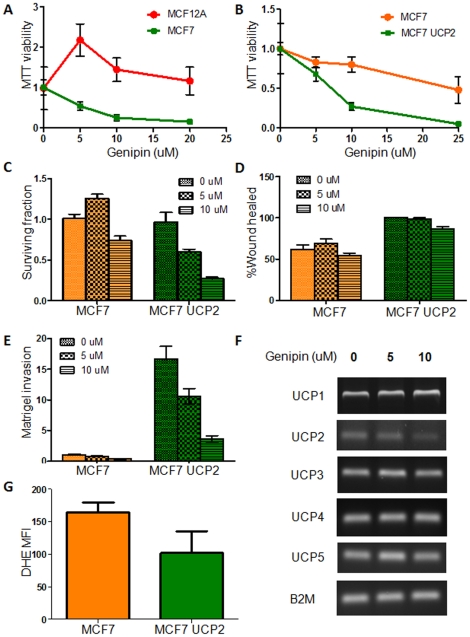
Genipin suppression of tumor promoting properties of UCP2. (A) MTT reduction of MCF12A and MCF7 cells after 48 h of genipin treatment. (B) MTT reduction after 48 h of genipin treatment demonstrating the increased sensitivity to genipin treatment of UCP2 over-expressing cells. (C) Clonogenic survival of UCP2 over-expressing cells treated with different concentration of genipin. (D) Wound healing assay with different concentration of genipin in the UCP2 over-expressing cells. (E) Matrigel invasion of MCF7 cells with different concentration of genipin in the UCP2 over-expressing cells. (F) UCPs expression after the genipin treatment in UCP2 over-expressing cells. (G) DHE oxidation of the MCF7 cells treated with genipin (10 uM) for 1 h.

Genipin is a small molecule derived from the gardenia plant [60]. Genipin inhibits UCP2-mediated proton leak and has been shown to reverse obesity, as well as high glucose-induced beta cell dysfunction in isolated pancreatic islets [53]. We hypothesized that genipin could suppress the tumor promoting property of UCP2 by inhibiting its expression and function. Treatment of MCF7 breast cancer cells with genipin decreased cell viability (with an IC_50_ of about 7 uM), whereas the genipin had little or no affect on the proliferation of MCF12A cells (Figure 5A). This is of interest because MCF7 cells express a significantly higher level of UCP2 protein than MCF12A cells. Next, we tested whether genipin’s effect was specific to UCP2 over-expression. We measured genipin’s effect on cell proliferation and the clonogenic survival of cells over-expressing UCP2. Our analyses revealed a concentration-dependent effect of genipin on cell proliferation and cell survival (Figure 5B–C). UCP2 over-expressing cells were much more sensitive to genipin treatment than control cells (Fig. 5B–C). Genipin treatment reduced migration in the wound-healing assay and significantly inhibited matrigel invasion of UCP2 over-expressing cells (Figures 5D, E). Interestingly, genipin treatment led to down-regulation of UCP2. UCP1, 3 4 and 5 were unaffected (Figure 5F). DHE oxidation (a measure of reactive oxygen species, mainly superoxide) was also decreased considerably with the genipin treatment (Figure 5G). These studies suggest that genipin inhibits the tumor promoting properties of UCP2 over-expressing cells, and this effect is mediated by down-regulation of UCP2.

### SMAD4 regulates UCP2 gene expression

**Figure 6:**
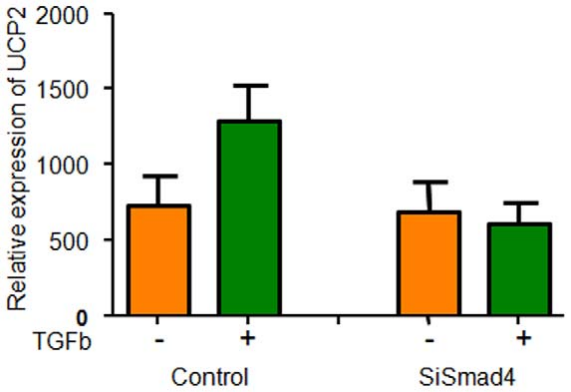
SMAD4 regulation of UCP2 gene expression. UCP2 is down-regulated by silencing Smad4 in the presence of TGFβ (see text for further detail).

Since UCP2 is over-expressed in a variety of cancers, we wished to identify a transcriptional regulator of UCP2. An analysis of the transcription factor binding sites of the UCP2 promoter revealed seven putative SMAD binding sites (−1179; −1378; −1445; −1506; −2005; −2401; −2738 relative to the first exon of UCP2). In order to understand the regulatory process this transcription factor has on UCP2, Smad4 was down-regulated by siRNA in mouse mammary epithelial NMuMG cells and UCP2 expression was assessed in the cells before and after treatment with TGF-β1. Microarray analysis was performed on duplicate samples of control and siRNA-Smad4 transfected cells. In the control cells, TGF-β1 up-regulated UCP2 transcript levels, whereas in the Smad4 knock-down cells, this regulation was abrogated (Figure 6). Two other UCP homolog genes, UCP1 and UCP3, were expressed at significantly lower levels and were not regulated by TGF-β1 (data not shown). We conclude that TGF-β1 *via* a Smad-dependent mechanism regulates UCP2 expression.

## Discussion

We took a novel strategy to identify genes responsive to irreversible injury to OXPHOS, as described by Warburg many years ago [9]. Our study revealed a similar pattern of gene-expression changes in rho^0^ epithelial cells, breast cancer cell lines and primary breast tumors. Our study identified that 37 genes were up-regulated and 30 genes were down-regulated in both rho^0^ and in cancer cell lines. These genes play important roles in transport, signal transduction, DNA replication, base excision repair, translation, apoptosis and electron transport. Among the up-regulated genes, UCP2’s expression was predominantly higher in rho^0^ epithelial cells, cancer cell lines and primary tumors. UCP2 belongs to a family of mitochondrial uncoupling proteins and is involved in dissipating the proton gradient across the mitochondrial membrane. The UCP family contains five members [53]–[60]. Among the five, UCP1 functions as a thermogenic protein in brown adipocytes [54]. UCP2 is ubiquitously expressed and protects cells from oxidative stress [55]. UCP2 also decreases glucose-induced hormone secretion in pancreatic islets and neurons [56]. Several reports also suggest UCP2 and 3 regulate fatty acid oxidation and mitochondrial calcium uniporter [57]. The other two members, UCP4 and UCP5, are expressed in a tissue-specific manner [58]. UCP4 and 5 reduce mitochondrial membrane potential [58]. Our study revealed a higher expression of UCP1, 4 and 5 in rho^0^ cells and cancer cell lines. However, the expression levels of UCP1, 3, 4 and 5 (few-fold) were insignificant when compared to UCP2 (several-hundred-fold). From this group, we chose UCP1 for its expression in primary tumors (breast and ovarian). UCP1 expression was increased in a small number (∼30%) of breast and ovarian tumors. In contrast, UCP2 was over-expressed in more than 80% of breast and ovarian tumors. Furthermore, UCP2 over-expression was found in other cancers, including leukemia, bladder, esophagus, testicular, colorectal, kidney, lung, pancreatic and prostate cancers. These studies suggest that UCP2 over-expression is involved in the development of a variety of cancer types and that UCP2 can function as a potential diagnostic marker associated with the Warburg Effect, described as a hallmark of cancers.

We demonstrate that UCP2 is transcriptionally regulated by SMAD4. UCP2 is regulated both at the transcriptional and translational levels. Sirt1 is described as a negative regulator of UCP2 transcription *via* regulatory proteins such as peroxisome proliferator-activated receptors (PPARs), forkhead transcription factors and sterol regulatory element-binding protein-1c (SREBP-1c) [59]. The UCP2 gene contains a short open reading frame (ORF1) in exon 2 that potentially encodes a putative peptide of 36 amino acids and inhibits translation of UCP2 mRNA [60]. A recent study in muscle cells suggests a microRNA (miR-133a) mediated regulation of UCP2 [61]. UCP2 protein is highly unstable and has a half-life of 30 min. UCP2 protein is rapidly turned over by the cytosolic proteasome [56]. The study presented in this paper suggests that UCP2 expression was up to 800-fold higher in primary breast tumors when compared with paired normal breast tissues. A similarly high level of expression was also detected in breast cancer cell lines. It is unclear how UCP2 expression is increased to such a high level transcriptionally. At the protein level, UCP2 expression in cancer cells was not as high, suggesting that both transcriptional and post-transcriptional regulation may play a role in tumorigenesis.

Very few studies have addressed the role of UCP2 in tumorigenesis. Studies suggest that UCP2 over-expression may protect cells from apoptosis [62]. UCP2 acts as a sensor of mitochondrial oxidative stress and is activated by ROS. The higher expression of UCP2 had been reported as cytoprotective by the negative regulation of mitochondrial ROS production. UCP2 function is an important component of local feedback mechanisms controlling the production of mitochondrial ROS [63]. UCP2 expression increases in response to the mitochondrial oxidative stress caused by electron transport chain inhibitors (Rotenone, antimycin A, DETC) [64]. An increase in ROS production is also reported in UCP2 knockout mice. Over-expression of UCP2 in colon cancer cells also inhibits ROS accumulation and apoptosis after exposure to chemotherapeutic agents [65]. It is likely that UCP2 over-expression also inhibits ROS accumulation in rho^0^ cells, otherwise mitochondrial defect may increase ROS production and trigger cell death.

UCP2 acts as a metabolic regulator. UCP2 was initially identified as the gene linked to obesity and hyperinsulinemia [38], [66]. Over-expression of UCP2 in isolated islets brings down glucose-stimulated insulin secretion [67]. Similarly, UCP2 knockout mice had shown increased glucose-stimulated insulin secretion [68]. UCP2 negatively regulates insulin secretion by systematic uncoupling, which in turns brings down the proton-motive force and the ATP:ADP ratio in the cytosol. Leukemia cells cultured on bone marrow-derived stromal feeder layers increase the expression of UCP2 and decrease the entry of pyruvate into the Krebs cycle [69]. Thus, increased UCP2 over-expression can directly contribute to the Warburg Phenotype. UCP2 is proposed to have activity as a uniporter for pyruvate [70]. As a result, UCP2 can promote pyruvate efflux from mitochondria and restrict glucose availability for respiration. This may increase the high rate of glycolysis in cancer cells [70]. Our studies suggest a novel function of UCP2. UCP2 is thought to function by lowering mitochondrial membrane potential to reduce oxidative stress that results from a hyperpolarized mitochondrial membrane. The up-regulation of UCP2 in rho^0^ cells, which are grossly impaired in mitochondrial function, suggests that the uncoupling function of UCP2 has a limited role. In rho^0^ cells the mitochondrial membrane potential is maintained by ATP hydrolysis by the ATP synthase. The ATP hydrolytic function of ATP synthase (Complex V) is maintained in the absence of mtDNA-encoded proteins. Thus, bioenergetically the uncoupling will cost more by depleting cytoplasmic ATP.

We provide evidence that UCP2 is over-expressed in a variety of tumors derived from different organs. A recent study also supports our findings in breast cancer [71]. To establish a cause-and-effect relationship between UCP2 over-expression and tumor development, we expressed UCP2 in MCF7 cells. Our studies suggest the tumor promoting function of UCP2 in vitro and in vivo in a mouse xenograft model. UCP2, when over-expressed, increases cell proliferation, migration and matrigel invasion and increases anchorage-independent growth. UCP2 over-expression also promotes growth of orthotopic tumors in vivo in athymic nude mice.

Genipin, a metabolite derived from the gardenia plant, is shown to be pharmacologically active against β-cell dysfunction. Genipin also inhibits UCP2-mediated proton leak [53]. We found that genipin treatment of UCP2 over-expressing cells decreases cell proliferation, clonogenic survival and matrigel invasion. In addition, genipin reduces cell proliferation of breast cancer cell line MCF7 but not the breast epithelial MCF12A cells. This effect of genipin was in part mediated by a decrease in mitochondrial membrane potential and down-regulation of UCP2 gene expression. Genipin did not significantly affect the expression of other UCPs. Our studies suggest that genipin can suppress tumor promoting function of UCP2.

Warburg hypothesized that injury to respiration was the underlying primary cause of tumorigenesis [14]. In his seminal 1956 article, Warburg discussed Feodor Lynen’s suggestion reported 14 years earlier that mitochondria in cancer cells could be uncoupled [72], [73], [74]. Accordingly, our studies described in this paper suggest that mitochondrial uncoupling protein UCP2 involved in uncoupling mitochondria plays an important role in breast, ovarian leukemia, bladder, esophagus, testicular, colorectal, kidney, pancreatic, lung and prostate cancers. Collectively, our studies suggest that i) UCP2 over-expression in tumors is a common phenomenon; ii) UCP2 over-expression promotes tumor development and iii) UCP2 over-expression can serve as a promising therapeutic target for treatment of breast and many other cancers. Furthermore, our genetic strategy revealed that cellular adaptation of cancer cells to mitochondrial defect may provide an opportunity to identify other gene targets that are vital to cancer cell survival and tumor development.

## Supporting Information

Table S1 **The list of nuclear genes encoding the mitochondrial proteome tiled on the MitoExpress.** The nuclear genes encoding the mitochondrial proteins were pooled and analyzed after a series of statistical and bioinformatic analyses. The corresponding probe sets of the genes were selected from the NetAffx. The standard probe sets for 146 housekeeping genes as that of human expression chip HG-U133 were included for normalization and background correction.
(XLS)

Table S2 **The list of genes that are down-regulated in both cancer and rho^0^ cell lines compared to the parental MCF12A rho^**+**^.** The gene expression that was twofold or lower compared to the parental MCF12A rho^+^ cell line was considered to be down regulated.
(XLS)

Table S3 **The list of genes that are up-regulated in both rho^0^ and breast cancer cell lines compared to the parental MCF12A rho^**+**^.** The gene expression that was twofold or higher compared to the parental MCF12A rho^+^ cell line was considered to be up regulated.
(XLS)
